# Renin‐angiotensin aldosterone profile before and after angiotensin‐converting enzyme‐inhibitor administration in dogs with angiotensin‐converting enzyme gene polymorphism

**DOI:** 10.1111/jvim.15746

**Published:** 2020-02-29

**Authors:** Darcy Adin, Clarke Atkins, Oliver Domenig, Teresa DeFrancesco, Bruce Keene, Sandra Tou, Joshua A. Stern, Kathryn M. Meurs

**Affiliations:** ^1^ College of Veterinary Medicine University of Florida Gainesville Florida; ^2^ College of Veterinary Medicine, North Carolina State University Raleigh North Carolina; ^3^ Attoquant Diagnostics Vienna Austria; ^4^ Department of Medicine and Epidemiology School of Veterinary Medicine, University of California Davis California

**Keywords:** ACE‐inhibitor, enalapril, genotype, pharmacogenetic, pharmacogenomic

## Abstract

**Background:**

An angiotensin‐converting enzyme (ACE) gene polymorphism occurs in dogs; however, functional importance is not well studied.

**Hypothesis:**

We hypothesized that dogs with the polymorphism would show alternative renin‐angiotensin aldosterone system (RAAS) pathway activation and classical RAAS pathway suppression before and after ACE‐inhibitor administration, as compared to dogs without the polymorphism that would show this pattern only after ACE‐inhibitor administration.

**Animals:**

Twenty‐one dogs with mitral valve disease that were genotyped for the ACE gene polymorphism.

**Methods:**

This retrospective study utilized stored samples from 8 ACE gene polymorphism‐negative (PN) dogs and 13 ACE gene polymorphism‐positive (PP) dogs before and after enalapril administration. Equilibrium analysis was performed to evaluate serum RAAS metabolites and enzyme activities. Results were compared before and after enalapril, and between groups.

**Results:**

The classical RAAS pathway was suppressed and the alternative RAAS pathway was enhanced for both genotypes after administration of enalapril, with no differences before enalapril administration. Aldosterone breakthrough occurred in both PN (38%) and PP (54%) dogs despite angiotensin II suppression. Aldosterone was significantly higher (*P* = .02) in ACE gene PP dogs (median, 92.17 pM; IQR, 21.85‐184.70) compared to ACE gene PN dogs (median, 15.91 pM; IQR, <15.00‐33.92) after enalapril.

**Conclusions and Clinical Importance:**

The ACE gene polymorphism did not alter baseline RAAS activity. Aldosterone breatkthrough in some dogs suggests nonangiotensin mediated aldosterone production that might be negatively influenced by genotype. These results support the use of aldosterone receptor antagonists with ACE‐inhibitors when RAAS inhibition is indicated for dogs, especially those positive for the ACE gene polymorphism.

**Abbreviations**
AA2: aldosterone to angiotensin II ratio
ABT: aldosterone breakthrough
ACE: angiotensin‐converting enzyme
ACE‐S: angiotensin‐converting enzyme activity marker
PCR: polymerase chain reaction
PN: polymorphism negative
PP: polymorphism positive
PRA‐S: plasma renin activity marker
RAAS: renin‐angiotensin aldosterone system

## 1 INTRODUCTION

The classical renin‐angiotensin aldosterone system (RAAS) is the neurohormonal cascade initiated by the release of renin that cleaves angiotensin I from angiotensinogen, followed by the conversion of angiotensin I to angiotensin II by the angiotensin‐converting enzyme (ACE) and production of angiotensin II metabolites (angiotensin III and angiotensin IV) by aminopeptidates, and ending in the stimulation of aldosterone release from the adrenal glands.^1^ Angiotensin II is the major stimulator of aldosterone synthesis and release from the adrenal cortex, although hyperkalemia and adrenocorticotrophic stimulating hormone can also induce aldosterone release.^2^, ^3^ The RAAS is activated in dogs with advanced heart disease, and contributes to disease progression and clinical signs.^1^, ^4^, ^5^, ^6^, ^7^ The major end‐metabolites of the classical RAAS are angiotensin II and aldosterone, both of which directly or indirectly mediate sodium and water retention, vasoconstriction, and pathological remodeling of cardiac, vascular, and renal tissues.^1^, ^4^, ^6^, ^7^ Recent reports describe an alternative RAAS pathway mediated by ACE2, prolyl‐carboxy‐peptidase 16, prolyl‐endo‐peptidase, and neprilysin, with production of the metabolites angiotensin 1‐9, angiotensin 1‐7 and angiotensin 1‐5 in people and dogs.^8^, ^9^, ^10^, ^11^ Additional metabolites within this cascade have also been discovered including angiotensin 2‐10, angiotensin 2‐7, and angiotensin 3‐7.^8^, ^10^, ^11^ Whereas the functional importance of some metabolites is not yet known, angiotensin 1‐7 has counterbalancing vasodilatory and natriuretic properties.^10^

Angiotensin‐converting enzyme‐inhibitors prevent the conversion of angiotensin I to angiotensin II, and thereby mitigate the maladaptive effects of these neurohormones through reduced formation of angiotensin II and angiotensin‐II‐driven aldosterone production.^1^ Polymorphisms of the ACE gene are recognized in human beings and dogs, which could influence the therapeutic strategies and outcome of diseases in which the RAAS plays a pathogenic role.^12^, ^13^, ^14^ Directly measured ACE activity is lower in dogs that are homozygous for a single base pair ACE gene polymorphism compared to dogs without the polymorphism; however, ACE activity after administration of ACE‐inhibitors is similarly suppressed regardless of genotype.^13^, ^14^ The functional effects and clinical importance of these findings are not well understood.

Although the RAAS has historically been challenging to investigate, renin‐angiotensin system equilibrium analysis has emerged as a novel technique that allows for comprehensive evaluation of this complex neurohormonal system. Catalyzing enzymes other than ACE (such as ACE2, neprilysin, chymase, and aminopeptidases) mediate production of RAAS metabolites in this pathway, and recent publications have begun to shed light on blood and tissue activities in dogs.^8^, ^9^, ^10^, ^11^ This methodology has shown that ACE‐inhibitors not only suppress angiotensin II formation, but also increase levels of the beneficial peptide hormone angiotensin 1‐7, which is known to be metabolized to angiotensin 1‐5 by the N‐domain of ACE.^15^ We sought to use equilibrium analysis to further investigate the functional effects of the ACE gene polymorphism at 9:11507816:G>A in dogs. Based on previous results showing reduced ACE activity in dogs positive for the ACE gene polymorphism,^13^, ^14^ we hypothesized that polymorphism‐positive dogs would show activation of the alternative RAAS pathway and suppression of the classical RAAS pathway both before and after administration of enalapril, as compared to polymorphism‐negative dogs that would show this pattern only after ACE‐inhibitor administration.

## 2 METHODS

This study was approved by the Institutional Animal Care and Use Committee at North Carolina State University College of Veterinary Medicine, and client consent was obtained. Dogs diagnosed with myxomatous mitral valve disease were included if they had not received cardiac medications, had at least a grade 4/6 left apical systolic murmur, and cardiac enlargement as determined by a radiographic vertebral heart scale of at least 11.^16^ Dogs with other systemic diseases as evident from routine chemistry panel or history were excluded. This study utilized samples stored from dogs meeting inclusion criteria that were previously genotyped for the ACE gene polymorphism at 9:11507816:G>A, 19 of which were previously reported.^14^ Biochemistry results, blood pressure, and ACE activity determined by radioenzymatic activity have been previously reported.^14^ Methodology for sample collection is summarized later.

Three milliliters of blood were obtained by peripheral venipuncture at the initial examination. One milliliter of blood was placed into an EDTA tube for genotyping. Two milliliters of blood were placed into a no‐additive tube. After centrifugation, the serum was removed and frozen at −80°. After the initial evaluation, all dogs were prescribed 0.5 mg/kg of enalapril orally every 12 hours, and repeat blood sampling to obtain serum was performed after 2 weeks of medication. Serum samples were frozen at −80° until overnight shipping on dry ice to the laboratory for batch analysis of RAAS metabolites as described later.

### 2.1 Genotyping

The previously validated primers (forward, 5′ TCAGCTCCATGCAATCCATA 3′; reverse 5′ CCCCTTGCCCTATCTGTAAA 3′) were used to carry out standard PCR amplification.^14^ Sequencing was visually inspected using commercially available software to identify the presence or absence of the known canine ACE gene polymorphism at 9:11507816:G>A, and if present, the genotypes (heterozygous or homozygous). Dogs that were negative for the ACE gene polymorphism were included in the control group (ACE gene polymorphism‐negative; PN; wildtype) and dogs that were either heterozygous or homozygous for the ACE gene polymorphism were included in the ACE polymorphism‐positive (PP; variant) group.

### 2.2 Equilibrium analysis of RAAS components

The equilibrium concentrations of 6 different RAAS angiotensin peptide metabolites and aldosterone in canine serum samples were quantified by liquid chromatography‐mass spectrometry/mass‐spectroscopy performed at a service provider laboratory (Attoquant Diagnostics, Vienna, Austria), using previously validated and described methods.^8^, ^10^, ^17^ Briefly, serum conditioning for equilibrium analysis was performed at 37°C followed by stabilization through the addition of an enzyme inhibitor cocktail (Waters, Milford, Massachusetts), as described previously.^8^, ^10^, ^17^ Previous results have shown similar qualitative outcomes when comparing the quantification of circulating (stabilized immediately at blood drawing) and equilibrium angiotensin peptide levels.^8^, ^10^, ^17^ Stabilized equilibrated serum samples were further spiked with stable isotope labeled internal standards for each angiotensin metabolite as well as with the deuterated internal standard for aldosterone (aldosterone D4) at a concentration of 200 pg/mL. The samples then underwent C‐18‐based solid‐phase‐extraction and were subjected to liquid chromatography‐mass spectrometry/mass‐spectroscopy analysis using a reversed‐phase analytical column operating in line with a Xevo TQ‐S triple quadruple mass spectrometer. Internal standards were used to correct for peptide and steroid recovery of the sample preparation procedure for each analyte in each individual sample. Analyte concentrations were reported in pM and are calculated considering the corresponding response factors determined in appropriate calibration curves in original sample matrix, on condition that integrated signals exceeded a signal‐to‐noise ratio of 10. The lower limit of quantification was 3.0 pM for angiotensin I, 2.0 pM for angiotensin II, 3.0 pM for angiotensin 1‐7, 2.0 pM for angiotensin 1‐5, 2.5 pM for angiotensin III, 2.0 pM for angiotensin IV and 15.0 pM for aldosterone.

The ratio of angiotensin II to angiotensin I was calculated as a marker for ACE activity (ACE‐S). Angiotensin I and angiotensin II were summed as a marker for plasma renin concentration (PRA‐S).^17^ The ratio of angiotensin 1‐5 to angiotensin 1‐7 (Ang 1‐5/Ang 1‐7) was calculated as an indicator of ACE activity driven by its N‐domain.^15^ The ratio of aldosterone to angiotensin II (AA2) was calculated as an indicator of adrenal responsiveness to angiotensin II stimulation of aldosterone release.^9^ Aldosterone breakthrough (ABT) was defined as any increase in serum aldosterone after enalapril compared to baseline (pre‐enalapril) aldosterone.^18^

### 2.3 Statistical analysis

Statistical analysis was performed using commercially available software (GraphPad Prism 8, San Diego, California). Data values that were below the lower limit of assay quantification were reported as half the lower limit for statistical analysis only.^19^, ^20^ Data sets were divided by polymorphism status as positive (PP) (including heterozygous and homozygous) or negative (PN), as well as by pre‐ or post‐ACE‐inhibitor (enalapril) treatment. Data were tested for normality using the D'Agostino and Pearson test and reported as medians and interquartile range because most were not normally distributed. Paired data (pre‐ and post‐enalapril data for each genotype group) were compared using Wilcoxin matched‐pairs signed rank test if data were nonparametric or using a 2‐way, paired *t* test if data were parametric. Unpaired data between groups (genotype comparison for pre‐enalapril and genotype comparison for post‐enalapril) were compared using Mann‐Whitney test if nonparametric or 2‐way, unpaired *t* test if parametric. Fisher's exact test was used to evaluate the effect of genotype on ABT. Significance was set at *P* < .05.

## 3 RESULTS

Eight PN dogs and 13 PP dogs were included. The mean age of PN dogs was 9.9 ± 3.8 (range, 5‐16) years, the mean weight was 9.0 ± 4.5 (range, 2.5‐18.9) kg, and the breeds were 3 Cavalier King Charles Spaniels, 2 Yorkshire Terriers, and 1 each of American Cocker Spaniel, Shih Tzu, and Toy Poodle. The mean age of PP dogs was 8.6 ± 1.8 (range, 7‐14) years, the mean weight was 11.1 ± 5.1 (range, 6.0‐22.0) kg, and the breeds were 8 Cavalier King Charles Spaniels, 2 Miniature Poodles and 1 each of mixed breed (Labradoodle), Norfolk Terrier, and Airedale. Polymorphism‐positive dogs included 8 dogs that were heterozygous for the polymorphism and 5 dogs that were homozygous for the polymorphism. There was no statistical difference in age (*P* = .3) or weight (*P* = .3) between PN and PP dogs. The mean (SD) time between assessments was 18.9 ± 9.9 days for PN dogs and 14.6 ± 3.0 days for PP dogs (*P* = .2).

### 3.1 Pre‐ and post‐enalapril comparisons

**Figure 1:**
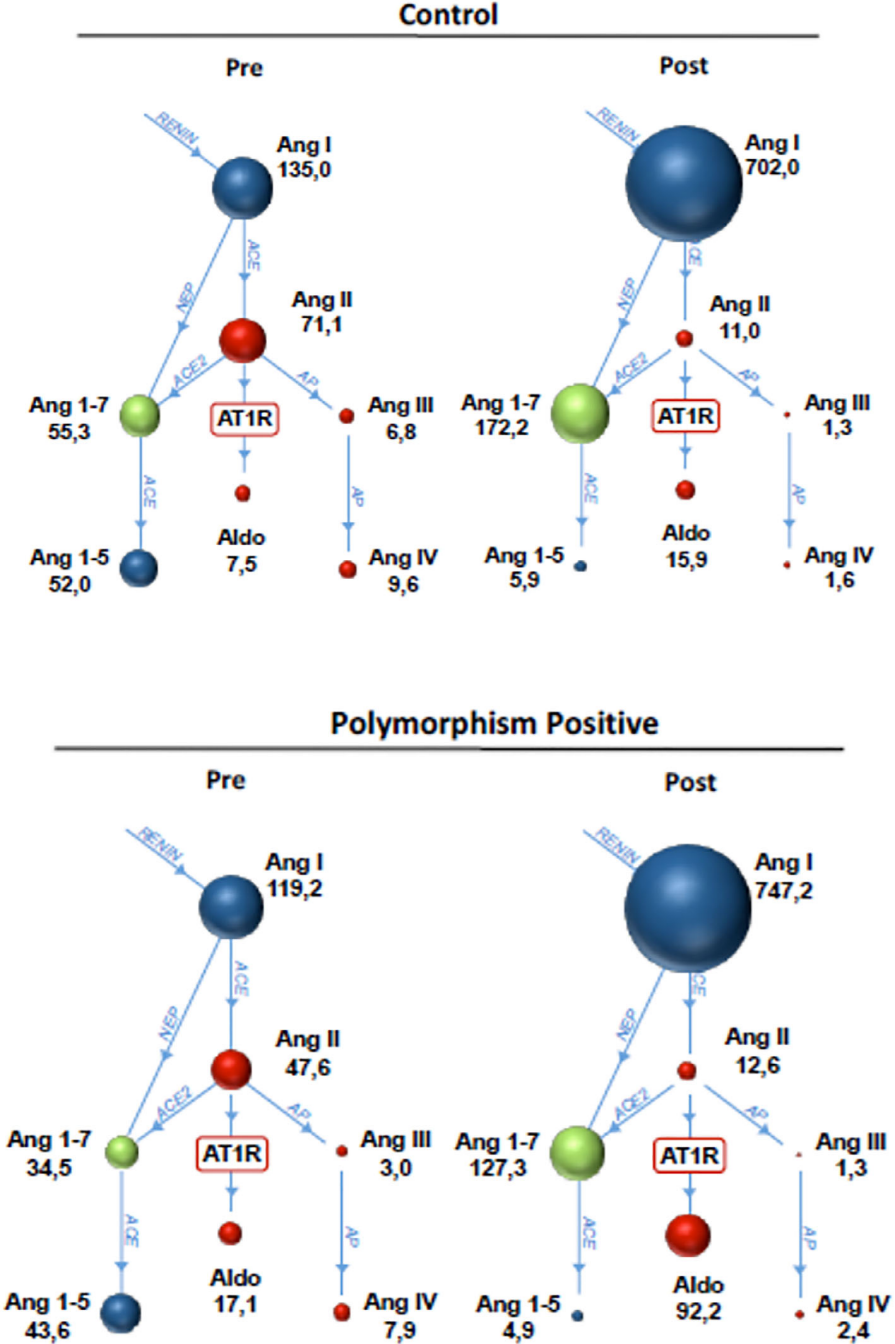
Renin‐angiotensin aldosterone system graphs in 8 control dogs that were negative for the ACE polymorphism and 13 dogs that were positive for the ACE polymorphism. Median values (pM) for each of the angiotensin metabolites and aldosterone are shown underneath each group before (pre) and after (post) enalapril. The size of the ball is proportional to the value. Values below the lower limit of quantification are shown as < the lowest reported value for each assay. Ang I, Angiotensin 1; Ang 1‐7, Angiotensin 1‐7; Ang II, Angiotensin II; Ang III, Angiotensin III; Ang 1‐5, Angiotensin 1‐5; Ang IV, Angiotensin IV; Aldo, Aldosterone; AT1R, Angiotensin II Receptor Type I; ACE, angiotensin converting enzyme; ACE2, angiotensin converting enzyme 2; AP, aminopeptidase; NEP, neprilysin

Polymorphism‐negative dogs showed a statistically significant increase in angiotensin I, angiotensin 1‐7, PRA‐S, and AA2, and a statistically significant decrease in angiotensin II, angiotensin 1‐5, ACE‐S and Ang 1‐5/Ang 1‐7 after treatment with enalapril. Three of 8 PN dogs (38%) demonstrated ABT (Table 1, Figure 1).

**Table 1:** Renin‐angiotensin aldosterone system (RAAS) metabolites and ratios, pre‐ and post‐enalapril, for ACE polymorphism negative dogs and ACE polymorphism positive dogs

	PN dogs (n = 8)		PP dogs (n = 13)		Pre‐enalapril versus post‐enalapril (*P* value)		PN versus PP (*P* value)	
Variable	Pre‐enalapril	Post‐enalapril	Pre‐enalapril	Post‐enalapril	PN	PP	Pre‐enalapril	Post‐enalapril
Ang I (pM)	134.2 (70.59‐150.6)	702.0 (432.3‐914.9)	119.2 (50.62‐156.60)	747.20 (353.8‐1068.0)	**0.0078**	**0.0003**	0.75	0.97
Ang II (pM)	55.14 (31.32‐83.18)	10.96 (7.86‐22.41)	47.56 (30.59‐89.14)	12.64 (3.38‐21.43)	**0.0078**	**0.0024**	0.97	0.84
Ang 1–7 (pM)	55.03 (41.30‐95.79)	172.2 (120.7‐277.5)	34.54 (17.16‐87.23)	127.30 (58.43‐248.90)	**0.039**	**0.0019**	0.37	0.72
Ang 1–5 (pM)	49.3 (40.33‐103.0)	5.86 (2.03‐17.07)	43.59 (21.04‐65.90)	4.90 (3.09‐10.65)	**0.0078**	**0.0005**	0.18	0.91
Ang III (pM)	5.19 (<2.50‐9.59)	<2.50 (<2.50‐ < 2.50)	3.00 (<2.50‐6.37)	<2.50 (<2.50‐2.03)	0.0625	**0.0039**	0.29	0.94
Ang IV (pM)	8.09 (3.89‐12.54)	<2.00 (<2.00‐4.16)	7.92 (<2.00‐11.56)	2.43 (<2.00‐4.02)	0.156	**0.0059**	0.71	0.84
Aldosterone (pM)	<15.00 (<15.00‐30.38)	15.91 (<15.00–33.92)	17.13 (<15.00‐77.77)	92.17 (21.85‐184.70)	1.00	0.32	0.19	**0.02**
AA2	0.2 (0.18‐0.76)	1.15 (0.65‐4.49)	0.56 (0.26‐2.03)	6.82 (1.72‐21.45)	**0.02**	**0.0002**	0.10	**0.02**
PRA‐S (pM)	173.4 (130.6‐212.0)	713.6 (436.6‐936.8)	161.1 (81.19‐262.6)	749.9 (355.9‐1087.0)	**0.016**	**0.0007**	0.75	0.92
ACE‐S	0.31 (0.24‐0.76)	0.02 (0.013‐0.030)	0.45 (0.37‐0.76)	0.020 (0.01‐0.03)	**0.0078**	**<0.0001**	0.35	0.94
Ang1‐5/Ang 1–7	1.05 (0.96‐1.25)	0.05 (0.01‐0.06)	0.98 (0.75‐1.32)	0.05 (0.03‐0.07)	**0.0078**	**0.0002**	0.66	0.56

*Note:* Values are shown as median and interquartile range. Statistically significant P values are bolded. Values below the lower limit of quantification are shown as < the lowest reported value for each assay.

Abbreviations: AA2, aldosterone to angiotensin II ratio; ACE‐S, angiotensin converting enzyme marker; Ang 1‐5, Angiotensin 1‐5; Ang 1‐5/Ang1‐7, angiotensin 1‐5:angiotensin 1‐7 ratio; Ang 1‐7, Angiotensin 1‐7; Ang I, Angiotensin 1; Ang II, Angiotensin II; Ang III, Angiotensin III; Ang IV, Angiotensin IV; PN, polymorphism negative; PP, polymorphism positive; PRA‐S, plasma renin activity marker.

Polymorphism‐positive dogs had a statistically significant increase in angiotensin I, angiotensin 1‐7, PRA‐S, and AA2, as well as a statistically significant decrease in angiotensin II, angiotensin I‐5, angiotensin III, angiotensin IV, ACE‐S and Ang 1‐5/Ang 1‐7 ratio after treatment with enalapril. Seven of 13 PP dogs (54%) demonstrated ABT.

### 3.2 Genotype comparisons

**Figure 2:**
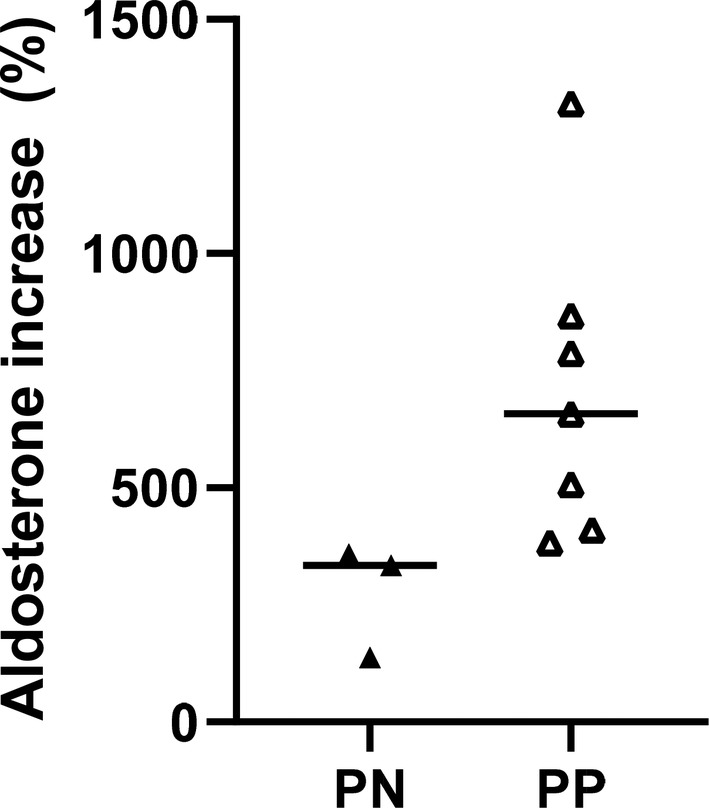
The percent increase in aldosterone after enalapril for dogs demonstrating aldosterone breakthrough was greater for ACE gene polymorphism positive dogs (PP) compared to ACE gene polymorphism negative dogs (PN) (*P* = .02)

No significant differences in the RAAS profile and enzyme activities were present between PN and PP dogs before enalapril treatment (Table 1, Figure 1). Post‐enalapril group comparisons showed significantly greater aldosterone concentrations and AA2 in PP dogs compared to PN dogs but the number of dogs that exhibited ABT was not different between genotypes (3 PN versus 7 PP; *P* = .6). When only the dogs that exhibited ABT were compared between genotypes, the percentage increase (PP median 658% compared to PN 334%; Figure 2) and absolute increase (PP 155 pM compared to PN 26 pM) in aldosterone was greater for PP dogs compared to PN dogs (*P* = .02).

## 4 DISCUSSION

This study failed to demonstrate different degrees of ACE activity based on genotype before ACE‐inhibitor treatment, in contrast to results of previous studies.^13^, ^14^ Although unexpected, this might be explained by the different methodologies used to quantify ACE activity between studies. Radioenzymatic assay was utilized in previous reports to directly measure the activity of the ACE enzyme.^13^, ^14^ The activity of ACE in the current study was determined indirectly using the turnover of angiotensin I to angiotensin II, measured by equilibrium analysis. Theoretically this approach could incorporate the contribution of other enzymes to angiotensin II formation and degradation, such as aminopeptidases and chymase that could be altered in PP dogs to maintain a stable angiotensin II level. Alternatively, the affinity of the ACE enzyme for the substrate used in the radioenzymatic assay may be affected by the presence of the ACE gene polymorphism, which could affect results in vitro. Additionally, the overall degree of RAAS activation before enalapril administration was not different between genotypes, suggesting that the functional effects of the polymorphism on angiotensin II production in the whole animal may be counterbalanced by upregulation of other enzymes before administration of ACE‐inhibitors.

This study also demonstrated a significantly greater aldosterone concentration and AA2 in ACE gene PP dogs, compared to PN dogs after treatment with the ACE‐inhibitor, enalapril. The remainder of the classical and alternative RAAS pathway components were not different based on genotype at baseline and after enalapril treatment. Suppression of the classical RAAS pathway and enhancement of the alternative RAAS pathway with the administration of enalapril was explained by inhibition of the ACE enzyme; however, with the exception of aldosterone and AA2, we did not find differences in the cascade based on genotype. The finding of greater post‐enalapril aldosterone and AA2 ratio (the latter supports aldosterone production independent of angiotensin II) in PP dogs was unexpected and potentially clinically important, as it provides a potential explanation for ABT in dogs.

The RAAS profile in *both* genotype groups showed significant suppression of angiotensin II formation as a result of approximately 2‐weeks’ administration of an ACE‐inhibitor. This finding, presumed to be secondary to ACE inhibition, was accompanied by expected increases in angiotensin I, PRA‐S (enhanced renal renin secretion because of negative feedback of lower angiotensin II), suppression of classical RAAS metabolites (angiotensin III and IV) and enhancement of the alternative RAAS metabolite angiotensin 1‐7. The Ang 1‐5/Ang 1‐7 ratio significantly decreased with enalapril treatment, and this supports both ACE‐inhibitor‐induced production and stabilization of the beneficial angiotensin 1‐7 product (ACE degrades angiotensin 1‐7 into angiotensin 1‐5; Figure 1). Promotion of angiotensin 1‐7 (predominantly through neprilysin conversion of angiotensin I to angiotensin 1‐7) is believed to mediate ACE‐inhibitor benefits of vasodilation and natriuresis.^8^ Alternate RAAS metabolite production did not differ based on genotype.

The RAAS profile of some dogs in this study after treatment with enalapril is consistent with ABT; however, this importantly *does not* appear to be mediated by angiotensin II that was appropriately suppressed with enalapril treatment. Aldosterone breakthrough is the term used to describe inadequate or temporary suppression of aldosterone, despite the administration of appropriate doses of ACE‐inhibitors.^21^ The phenomenon of ABT has been shown to occur in both people and dogs.^18^, ^21^ Underlying mechanisms of ABT may include upward drift of ACE activity in the face of chronic RAAS‐suppressive treatment or ACE‐independent production of either angiotensin II or aldosterone.^18^, ^21^, ^22^ We found *no* evidence of inadequate ACE suppression in this study. The finding of high aldosterone in conjunction with suppressed angiotensin II formation in some dogs in this study suggests that ABT is occurring independent of angiotensin II. A multitude of peptides serve as substrates for ACE, and the accumulation of these peptides as a result of ACE‐inhibition could be contributing to the formation of aldosterone through other pathways.^23^ These findings are important because they demonstrate the efficacy of enalapril in suppressing angiotensin II formation, but also show ABT and indicate that multimodal RAAS suppression may be indicated to address modulation of other pathways by ACE‐inhibitors. Angiotensin II and aldosterone, directly or indirectly, contribute to sodium and fluid retention, and both promote inflammation, cell death, and fibrosis.^1^ These results provide further evidence of ABT and indicate that the combination of mineralocorticoid receptor antagonists (eg, spironolactone or eplerenone) and inhibition of angiotensin II formation or binding (ACE‐inhibitors or angiotensin receptor blockers, respectively) is often necessary to provide comprehensive RAAS inhibition.^18^, ^21^, ^24^ Additional study is required to elucidate other pathways that may be impacted by RAAS inhibitors.

Additionally, we found that the magnitude of ABT was greater in PP dogs compared to PN dogs despite suppression of angiotensin II formation with enalapril in both groups. This was also reflected in significantly greater AA2 ratio in PP dogs after enalapril treatment, indicating that the high aldosterone was independent of angiotensin II. This finding suggests an influence of the ACE gene polymorphism on an, as of yet unidentified angiotensin II‐independent pathway of aldosterone stimulation. In addition to the many possible substrates for ACE, there is recent evidence that numerous non‐angiotensin‐mediated, nonelectrolyte factors can control aldosterone secretion from the adrenal cortex.^22^ These paracrine regulatory factors include bioactive signals released from mast cells (eg, serotonin), nerve fibers (eg, catecholamines), chromaffin cells, adipocytes (eg, leptin), vascular endothelial cells (eg, endothelin 1), and steroidogenic cells (eg, prostaglandin E2).^22^ The influence of genotype in this setting requires further study to explore the impact of this polymorphism on dogs receiving ACE inhibition.

The results of this study provide mechanistic insight into the phenomenon of ABT and hold clinical implications for dogs with advanced heart disease. Although ABT occurs in a substantial minority (30%‐40%) of people and dogs receiving ACE‐inhibitors, predicting which patients are or will be affected is elusive.^18^, ^21^ Aldosterone breakthrough occurring with short‐term ACE‐inhibitor monotherapy in this study was independent of angiotensin II, and the ACE gene polymorphism appeared to negatively influence aldosterone concentrations in this group of dogs with preclinical mitral valve disease. Dogs positive for the ACE gene polymorphism might be at greater risk for adverse effects associated with unopposed circulating aldosterone than dogs negative for the polymorphism, despite the effectiveness of enalapril in suppressing angiotensin II production in both groups. The genetic heterogeneity of the ACE gene and the nonuniversal occurrence of ABT in dogs could be an explanation for discordant outcome findings of previous studies reporting the long‐term effects of ACE‐inhibitors in dogs with heart disease.^25^, ^26^

This study is limited by the small study population, which could have affected the ability to find differences between groups for some parameters. We did not separately analyze dogs that were homozygous from those that were heterozygous for the ACE gene polymorphism because of small subject numbers. Future studies with larger numbers will be necessary to determine if there are RAAS profile differences in dogs with 1 or both abnormal alleles. Genotyping might also prove useful for determining subpopulations of dogs that would benefit the most from aldosterone antagonizing medications.

In conclusion, we demonstrated that ABT occurs independent of successful angiotensin II suppression with ACE‐inhibitor treatment in some dogs, and that the ACE gene polymorphism seems to negatively influence this suppression. Additionally, the presence of the polymorphism appears to not reduce overall RAAS activation in the untreated dog, possibly because of upregulation of compensatory enzymes that might serve to stabilize angiotensin II levels. These findings not only improve our understanding of the RAAS in dogs, but also show functional importance of the ACE gene polymorphism in canine patients. The results of this study support the use of aldosterone receptor antagonists in conjunction with ACE‐inhibitors in dogs requiring RAAS suppression.

## CONFLICT OF INTEREST DECLARATION

Dr. Adin has received funding from CEVA Animal Health. Dr. Atkins has received funding and has consulted for CEVA Animal Health, Boehringer‐Ingelheim, and Vetoquinol. Dr. Meurs has received funding from the Morris Animal Foundation for parts of this study. Dr. Domenig is employed by Attoquant Diagnostics.

## OFF‐LABEL ANTIMICROBIAL DECLARATION

Authors declare no off‐label use of antimicrobials.

## INSTITUTIONAL ANIMAL CARE AND USE COMMITTEE (IACUC) OR OTHER APPROVAL DECLARATION

This study was approved by the IACUC (#13‐103‐0) at North Carolina State University Veterinary Hospital and owner consent was obtained.

## HUMAN ETHICS APPROVAL DECLARATION

Authors declare human ethics approval was not needed for this study.
